# The implication of TET1, miR-200, and miR-494 expression with tumor formation in colorectal cancer: through targeting Wnt signaling

**DOI:** 10.1007/s11033-024-10060-3

**Published:** 2024-11-04

**Authors:** Raziye Tajali, Neda Zali, Fatemeh Naderi Noukabadi, Meysam Jalili, Morteza Valinezhad, Farnaz Ghasemian, Makan Cheraghpour, Sanaz Savabkar, Ehsan Nazemalhosseini Mojarad

**Affiliations:** 1 Basic and Molecular Epidemiology of Gastrointestinal Disorders Research Center, Research Institute for Gastroenterology and Liver Diseases, Shahid Beheshti University of Medical Sciences, Yeman St, Chamran Expressway, P.O. Box 19857-17411, Tehran, Iran; 2 Gastroenterology and Liver Diseases Research Centre, Research Institute for Gastroenterology and Liver Diseases, Shahid Beheshti University of Medical Sciences, Yeman Street, Chamran Expressway, P.O. Box: 19857-17411, Tehran, Iran; 3 Department of Surgery, Leiden University Medical Center, Leiden, Netherlands

**Keywords:** Colorectal cancer, Epigenetics, TET protein, Wnt pathway, 5-aza

## Abstract

**Objective:**

Colorectal cancer (CRC) is a diverse and multifaceted disease characterized by genetic and epigenetic changes that contribute to tumor initiation and progression. CRC pathophysiology has been linked to the deregulation of the Wnt signaling pathway and the ten-eleven translocation (TET) DNA demethylases. This study aimed to evaluate the expression level of selective miRNAs (miR-200 and miR-494), TET1, and Wnt1 in colorectal polyps, actual colorectal tumors, and normal adjacent tissues. We also evaluated the effect of 5-aza cytidine on the expression level of TET1 and wnt1 in the HT29 cell line.

**Materials and methods:**

In this study, we assessed TET1 and Wnt1 expression in 5-azacytidine-treated HT29 cells, a demethylating agent commonly used in cancer therapy. Additionally, we enrolled 114 individuals who underwent radical surgical colon resection, including 47 with cancerous tissues and 67 with polyps. We utilized qRT-PCR to measure miR-200, miR-494, TET1, and Wnt1 mRNA levels in colorectal polyps, actual colorectal tumors, and normal adjacent tissues.

**Results:**

Our study revealed that TET1 expression was notably lower in both polyps and CRC tissue compared to adjacent normal tissue, with higher TET1 expression in tumors than polyps. We also observed significant differences in miR-200 and miR-494 expression in tumor samples compared to adjacent normal tissue. Our in vitro experiments revealed that 5-azacytidine administration increased TET1 and decreased Wnt1 expression in CRC cell lines. This suggests that DNA-demethylating drugs may have a therapeutic role in modifying TET1 and Wnt signaling in the development of CRC.

**Conclusions:**

Overall, our findings shed light on the intricate interactions between TET1, Wnt1, and specific miRNAs in colorectal cancer (CRC) and their potential implications for diagnosis and treatment.

## Introduction

The complicated condition known as colorectal cancer (CRC) results from multi-step carcinogenesis due to the buildup of genetic and epigenetic alterations in the cells lining the rectum or colon. CRC exhibits molecular heterogeneity, and its development involves the gradual accumulation of morphological, genetic, and epigenetic alterations leading to the transition from normal colonic epithelial cells to adenocarcinoma [1]. The study of these epigenetic changes, which refer to changes in the DNA structure that do not change the underlying sequence of nucleotides but affect how the DNA is packaged and expressed, presents a significant intellectual challenge. The most extensively characterized epigenetic modification is methylation at the 5-carbon of cytosines, mainly in the context of CG dinucleotides [2]. DNA methylation modifies the attachment of transcription factors to their target sites on DNA, which may change the expression of downstream genes, even though the underlying molecular mechanisms of DNA methylation are not fully understood [2]. Ten-eleven translocation (TET) proteins are essential mediators of active DNA methylation in coordination with DNA methyltransferases (DNMTs) [3]. Members of the TET family (TET1-TET3) are involved in the demethylation process by acting as methylcytosine dioxygenases, catalyzing the conversion of 5-methylcytosine (5mC) to 5-hydroxymethylcytosine (5hmC) and formyl cytosine (5fC) to carboxyl cytosine (5caC) [4].

Furthermore, evidence suggests that TET proteins have an additional role in regulating cancer-related genes through mechanisms independent of their enzymatic activity. Down-regulation or dysfunction of TET1 is associated with cancer initiation, invasion, and metastasis. Certainly, TET1 is frequently lacking in various cancers [5]. Additionally, studies have indicated a down-regulation of TET1 in early-stage colon tumors, where the decreased expression of TET1 during the initiation of colon cancer suppresses the promoters of WNT pathway inhibitors, leading to a persistent activation of the WNT pathway [4]. The mechanisms by which TET family members function and exert their action in colorectal carcinogenesis and progression have not yet been clearly described.

MicroRNAs (miRNAs) are a category of non-coding RNAs that serve as crucial regulators of essential processes in the body. They connect to the 3’ untranslated region (UTR) of messenger RNAs and are considered critical controllers of gene expression. Disruptions in their function can result in the emergence of aberrant gene expression patterns, potentially contributing to disorders such as cancer. MiRNAs can act as tumor suppressors or oncogenes, playing roles in the pathogenesis of many cancers, such as CRC. A broad range of miRNAs are involved in colorectal carcinogenesis by regulating genes involved in cancer cell growth, proliferation, apoptosis, invasion, and metastasis [6]. Among these, it has been shown that miR-200b was down-regulated in CRC while miR-494 was up-regulated, associated with tumor-promoting [7, 8].

Wnt1 (wingless-related integration site) is a member of the WNT family and plays a role in activating the canonical WNT signaling pathway. It has been demonstrated that Wnt1 was able to induce resistance to treatment and inhibit apoptosis through stimulation of beta-catenin. In other words, it enhances the proliferation and migration of colorectal tumors which was associated with CRC development. Some microRNAs target the WNT/beta-catenin signaling, inhibiting the proliferation and invasion of CRC cells and inducing apoptosis [9]. Therefore, inhibiting Wnt1 may potentially suppress the growth of colorectal cancer through epigenetic modifications.

However, it is crucial to define the exact systems and interactions between TET1 and miRs with WNT1 in CRC, which will augment the understanding of the regulatory mechanisms of CRC. This study aimed to investigate the expression levels of miR200b, miR-494, TET1, and Wnt1 in cancerous tissues, polyps, and corresponding adjacent normal tissues from CRC patients. In addition, we evaluated the effect of DNA methyltransferase inhibitor 5-azacitidine (5-aza) on the expression level of TET1 and Wnt1 in the HT29 cell line.

## Materials and methods

### Cell culture and treatment

The HT29 human colon cancer cell line was purchased from the Pasteur Institute of Iran and cultured in RPMI 1640 medium (Gibco; Life Technologies, Carlsbad, CA, USA) supplemented with 10% fetal bovine serum (FBS; Sigma-Aldrich et al., USA) and 100 IU/ml of penicillin/streptomycin (Thermo et al., USA) under sterile conditions and maintained at 37 °C in an incubator under an atmosphere of 5% CO2. The medium was refreshed every two days, and cells were passaged once a week. For the 5-aza treatment experiments, cells were subjected to a precise range of concentrations of 5-aza (0.5, 1, 2.5, 5, 10, and 20 µM) for 24, 48, and 72 h. The 5-aza, sourced from Sigma (St. Louis, MO, USA), was carefully dissolved in culture medium and phosphate-buffered saline (PBS) [10].

### Cell viability assay

Cell cytotoxicity and IC50 dose for 5-aza were detected using the MTT colorimetric assay. Cells were seeded at a density of 5000 cells/well in a 96-well plate. After overnight incubation, the cells were treated in triplicate with different concentrations 5-aza (0.5, 1, 2.5, 5, 10, and 20 µM), and DMSO as the vehicle. Following a 72-hour incubation period, the medium was aspirated from the wells, and 20 µL of MTT solution (5 mg/mL in PBS) was introduced into each well, then incubated at 37º C, 5% CO_2_, for five hours. The formazan crystal was solubilized using 200 µL DMSO. The samples were read at 570 nm on a microplate reader (BioTek, USA).

### Patient samples

Colorectal polyps, actual colorectal tumors, and normal adjacent tissues were collected between June 2019 and March 2020 from a cohort of 114 patients who received radical surgical resection of the colon at the Department of Gastroenterology and Liver Diseases (RIGLD) of Shahid Beheshti University of Medical Science, Iran. Histopathological unchanged colonic mucosa located at least 10–20 cm away from the cancerous lesions was obtained from the same patients. The Age of CRC diagnosis ranged from 28 to 84 years (median: 64 years) (Table 1). Samples were instantaneously snap-frozen in liquid nitrogen and kept at − 80 °C until DNA and RNA isolation. Preoperative radiation or chemotherapy was not administered to any of the patients. Each and every participant in the study gave informed consent. The RIGLD ethics committee approved the study’s methods (Ethical Code of Education 1392/704). Written consent was obtained from all participants.

**Table 1:** Basic characteristics of subjects

Variable	*N*
Sex	
Male	53
Female	61
Age	
≤ 50	15
> 50	99
BMI	
9/24 − 5/18	59
9/29 − 25	48
9/34 − 30	7
Smoking	
Yes	7
No	107
Tumor	47
Polyp	67
Location	
Colon	105
Rectum	9
Blood pressure	
Yes	11
No	103
IBD	
Yes	10
No	104

**Table 2:** Details of the primer pairs used in this study

Gene or miRNA name	Primer	Sequences (5’→3’)
*TET1*	Forward	TCTTGTCCTCCCAAAGTGCT
	Reverse	TGCCTGTCATGCTGTCTT
*Wnt1*	Forward	GCCCAGGTTGTAATTGAAGC
	Reverse	TGAGAAAGTCCTGCCAGTTG
*GAPDH*	Forward	CCTTCATTGACCTCAACTACATG
	Reverse	TGGGATTTCCATTGATGACAAGC
miR-200b	Forward	AAGTAACCTCCAGAGCCC
	Reverse	GTGGGTCTCAGGATCGG
miR-494	Forward	CATAGCCCGTGAAACATACACG
	Reverse	GTGCAGGGTCCGAGGT
U6	Forward	TGACCTGAAACATACACGGGA
	Reverse	TATCGTTGTACTCCACTCCTTGAC

### RNA isolation, reverse transcription, and real-time quantitative polymerase chain reaction (qRT-PCR) analysis

Total RNA was extracted from tissue samples, HT29 cells, and 5-aza-treated HT29 cells utilized a Qiagen extraction kit (Germany) following the manufacturer’s protocol. Nanodrop spectrophotometer (Thermo Scientific, USA), ensuring accurate measurement and analysis. cDNA was synthesized from three µg of RNA using a First Strand cDNA Synthesis kit. Real-time PCR (RT-PCR) was conducted on a StepOnePlus Real-Time PCR System, employing TaqMan Gene Expression Assays for *TET1* and Wnt1 genes. PCR conditions included denaturation at 95 °C for 20 s, annealing at 60 °C for 20 s, and extension at 72 °C for 30 s over 40 cycles. PCR efficiencies were determined by linear regression slope analysis, with efficiencies ranging from 98 to 102%. All assays were performed in triplicate. The comparative 2^-ΔΔCT method was utilized to calculate the relative fold changes and normalizing to reference genes GAPDH and U6 [11]. Table 2 provides details on the characteristics of the designed primers.

### Measurement of miR-200b and miR-494 Expression Levels by qRT-PCR

The expression levels of miR-200b and miR-494 were assessed using qRT-PCR. Total RNA, including microRNA, was isolated from tissue samples and HT29 cells treated with 5-aza using the Qiagen miRNeasy Mini Kit (Germany) following the manufacturer’s protocol. cDNA was synthesized using the TaqMan MicroRNA Reverse Transcription Kit (Applied Biosystems, USA). qRT-PCR was performed on the StepOnePlus Real-Time PCR System with TaqMan MicroRNA Assays specific for miR-200b and miR-494. U6 small nuclear RNA was used as an internal control. The PCR conditions were as follows: 95 °C for 10 min, followed by 40 cycles of 95 °C for 15 s and 60 °C for 60 s. Relative expression levels were calculated using the comparative 2^-ΔΔCT method [11].

### Statistical analysis

Data analysis was conducted using GraphPad Prism version 8.4.3 (GraphPad Software, Inc., La Jolla, CA, USA). Two-tailed Student’s t-test or one-way analysis of variance followed by Dunnett’s multiple comparison test were used to assess statistical significance. Correlation was measured using SPSS using Pearson’s correlation coefficient (r). A significance level of *P* < 0.05 was used to determine statistical significance. The experiments were independently repeated at least three times, and the findings are shown as the mean ± standard deviation.

## Results and discussion

### DNA methylation and cancer development (Measurement of TET1 and Wnt1 gene expression level by qRT-PCR)

**Fig. 1:**
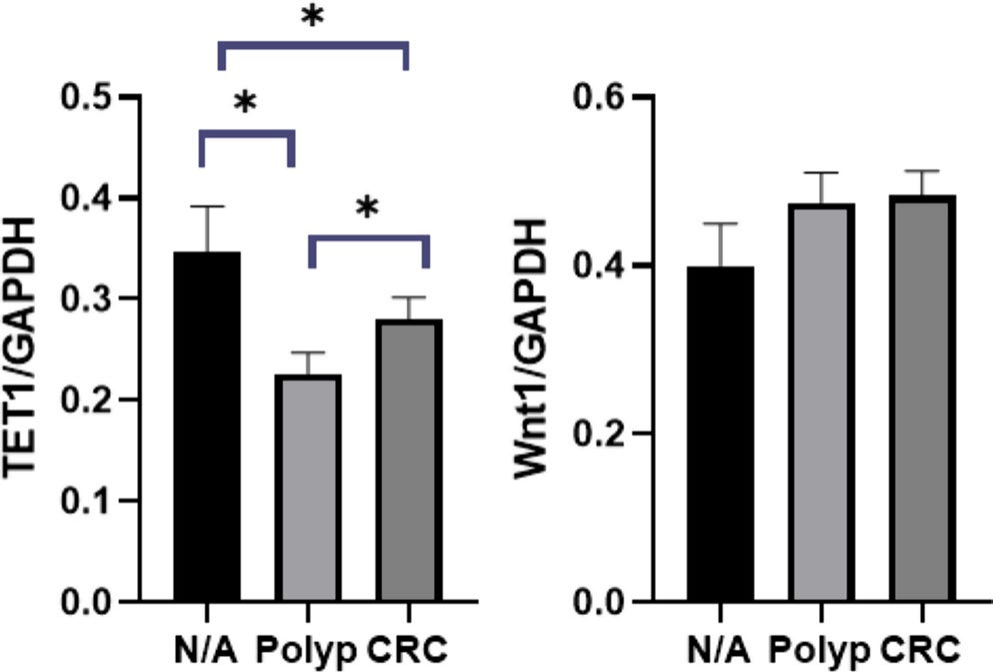
Real-time PCR of TET1 gene expression in the HT29 and 5-aza-HT29 cells. Significant decrease in the expression of the TET1 gene in polyp and CRC samples compared to adjacent normal tissue (A/N). The expression of the TET1 gene was found to be significantly higher in tumor samples compared to polyp tissues (B). There was no significant difference in the expression of the Wnt1 gene in polyp and CRC tissue. Data are shown after normalization to GAPDH (ns *P* > 0.05, *P ≤ 0.05)

Aberrant DNA methylation, a key characteristic of cancer cells, is essential for activating or inactivating tumor-related genes. Indeed, several genes are frequently aberrantly methylated at different steps in the sequence from polyp to CRC [12]. TET1 downregulation, commonly observed in CRC, can be used as a marker to improve cancer diagnosis, a prognostic biomarker, a predictive biomarker for therapy response, a potential therapeutic target, and an insight into the underlying biology of the disease. Previous studies have consistently reported reduced TET1 transcript levels in cancerous tissues [4]. However, TET1 expression in polyps has not been investigated in previous studies. In this study, we assessed the expression levels of the TET1 and Wnt1 genes in polyp and tumor samples, as well as their corresponding adjacent normal tissues. Our findings indicated a significant reduction in TET1 gene expression in polyp (*P* = 0.026) and colorectal cancer (CRC) samples (*P* = 0.046) when compared to the adjacent normal tissues. Moreover, it was discovered that tumor samples had substantially higher TET1 gene expression than polyp tissues (*P* = 0.040) (Fig. 1).

The Wnt signaling pathway is crucial for maintaining cellular homeostasis, and its dysregulation is common in many cancers, including CRC. TET1 regulates the Wnt pathway by targeting genes that act as upstream inhibitors of Wnt signaling, such as DKK and SFRP [1, 13]. These results suggest that TET can suppress the WNT pathway in cancer cells, preventing the initiation and progression of CRC. However, our results showed no significant variance in the expression of the *Wnt1* gene between polyp and adjacent normal tissues (*P* = 0.614) or between tumor and adjacent normal tissues (*P* = 0.311). Studies on Wnt1 expression in CRC tissues are challenging due to the contradictory results. While most studies have reported increased Wnt1 expression in CRC tissues compared to usual colorectal mucosa, highlighting its significant role in promoting tumor growth, invasion, metastasis, and chemotherapy resistance, some studies have observed a lack of expression. Our findings highlight the intricate relationship between DNA methylation, TET enzymes, and the Wnt signaling pathway in CRC. Aberrant DNA methylation is a hallmark of cancer, and TET1 plays a pivotal role in DNA demethylation, thereby regulating gene expression.

### 5-Azacytidine in CRC treatment (the effects of 5-Azacytidine on TET1 and Wnt1 gene expression)

Wang et al. discovered that the overexpression of TET1 inhibited cell proliferation, induced apoptosis, arrested cell cycle progression, hindered migration and invasion, and reduced activity of the Wnt/β-catenin signaling pathway and nuclear β-catenin expression. Additionally, it increased 5hmC levels while decreasing 5mC levels [13]. In addition, TET1 may have a role in the pharmacological action of a DNA methyltransferase (DNMT) inhibitor. Therefore, restoring TET1 function could be a practical therapeutic approach for CRC treatment [14]. In CRC, 5-aza suppresses DNA methylation, potentially altering the methylation state of genes involved in the Wnt signaling pathway and inhibiting Wnt1 production [15].

Our research investigated the potency of 5-azacytidine (5-aza) on HT29 cell growth and its impact on TET1 and Wnt1 gene expression. The MTT assay proved that 5-aza was highly effective in reducing HT29 cell viability at 24, 48, and 72 h, with an IC50 value of 1.25 µM. This dose-dependent reduction suggests the effectiveness of 5-aza in inhibiting CRC cell proliferation. This 5-aza reduced dose-dependently is proof of its efficiency in halting the proliferation of CRC cells.

**Fig. 2:**
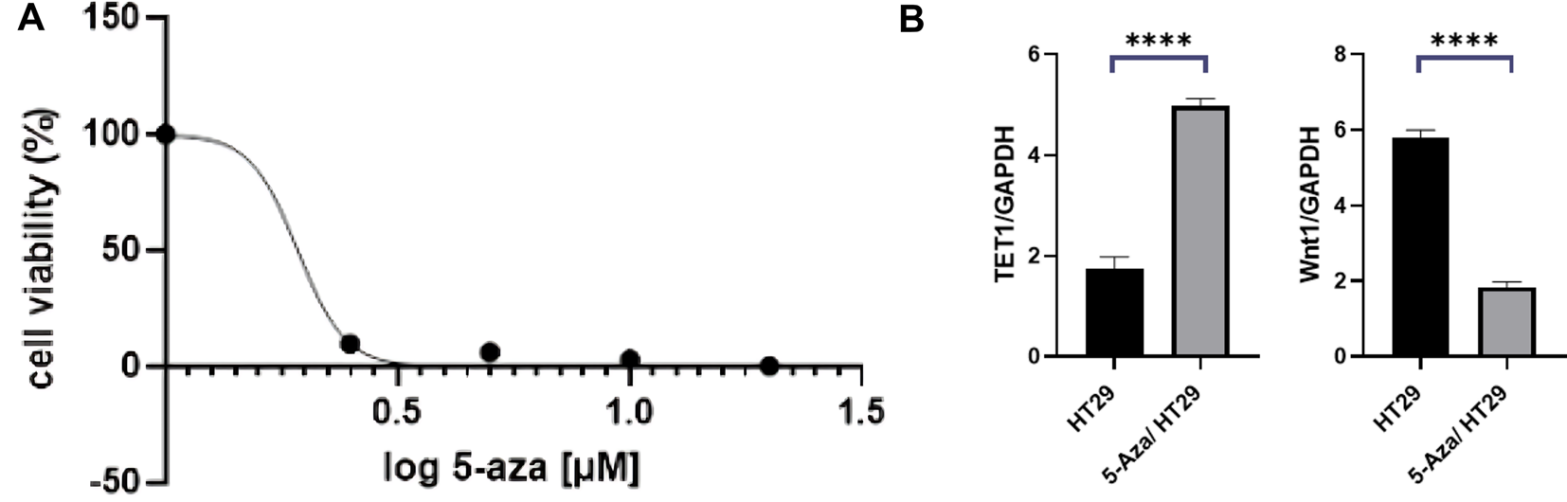
The effect of 5-aza treatment. **A**) 1. 5-aza treatment in the HT29 colon cancer cell line leads to cell viability reduction. Concentration–response curve was used to determine IC50 at a concentration of 1.25 µM, which indicated 50% growth inhibition. **B**) Real-time TET1 and Wnt1gene expression in the HT29 and 5-aza-HT29 cells. Comparing the effect of 72 h treatment with 5-aza on the expression of all three genes in the HT29 cells. All values are presented as the mean ± SE of three independent experiments in triplicate. * Denotes a statistically significant difference (*p* < 0.05) compared to the HT29 and control cells, respectively

To further explore the molecular mechanisms underlying this inhibitory effect, we evaluated the expression levels of TET1 and Wnt1 using qRT-PCR. The results showed a significant upregulation of TET1 expression in 5-aza-treated cells compared to control cells (*P* < 0.0001) [16]. This increase in TET1 expression suggests that 5-aza may promote active DNA demethylation, a process regulated by TET enzymes, which has been shown to play a critical role in gene regulation and cell differentiation. Conversely, 5-aza treatment significantly downregulated the expression of Wnt1 (*P* < 0.0001) [17] (Fig. 2). This indicates that regulating Wnt1 in CRC has multiple sides and can vary according to particular factors [15, 16].

### MicroRNAs in colorectal cancer (Measurement of miR-200b and miR-494 expression Level by qRT-PCR)

MicroRNA’ is a small RNA molecule that does not code for protein, but it regulates the expression of genes by complementary base pairing with one or more mRNAs. DNA methylation is an epigenetic alteration that significantly affects microRNA expression and frequently happens as CRC progresses [16]. The expression of miR-494 in CRC tissues was significantly increased compared to the adjunct tissues, which was associated with TET1 down-regulation in CRC tissues. miR-494 expression is inversely correlated with the adenomatous polyposis coli (APC) gene expression, and thus, miR-494 directly targets APC in CRC [17]. In addition, miR-494 influences the Wnt/β-catenin signaling pathway. An overabundance of miR-494 has been observed to stimulate this pathway by affecting APC, thereby promoting the growth of CRC cells [17]. Our results showed a significant increase of miR-494 expression in the CRC tissue samples compared to adjacent normal tissue (*P* = 0.025). Our data showed no significant upregulation in polyp compared to the adjacent samples. This may be attributed to the absence of distinct group separation among polyps in this study.

A study involving invasive human hepatocellular carcinoma tumors found that miR-494 can silence several miRNAs. By directly targeting TET1, this silencing mechanism prevents the demethylation of genomic DNA, thereby aiding in the start of tumor vascular invasion [16]. Therefore, it seems miR-494 promoted the growth of CRC cells by inhibiting TET1 expression and stimulating the Wnt/β-catenin signaling pathway.

**Fig. 3:**
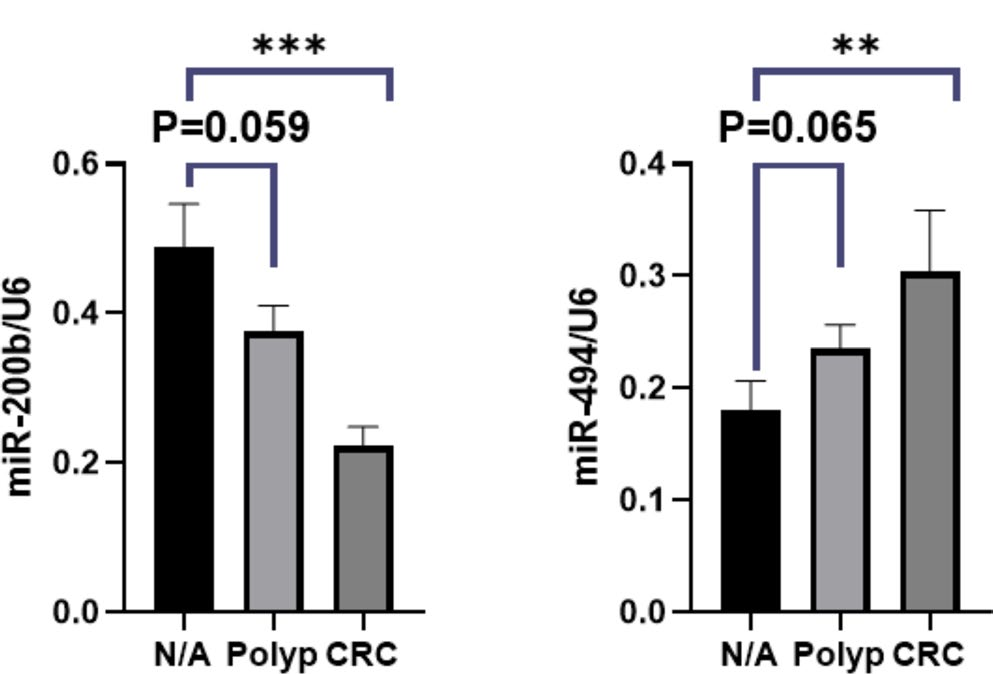
Real-time PCR of TET1 gene expression in the HT29 and 5-aza-HT29 cells. Significant decrease in the expression of the mir-200b CRC samples compared to adjacent normal tissue (A/N). The expression of the mir-494 was found to be significantly higher in CRC samples compared to adjacent normal. Data are shown after normalization to U6 (ns *P* > 0.05, *P ≤ 0.05, **P ≤ 0.01, ***P ≤ 0.001, ****P ≤ 0.0001)

**Fig. 4:**
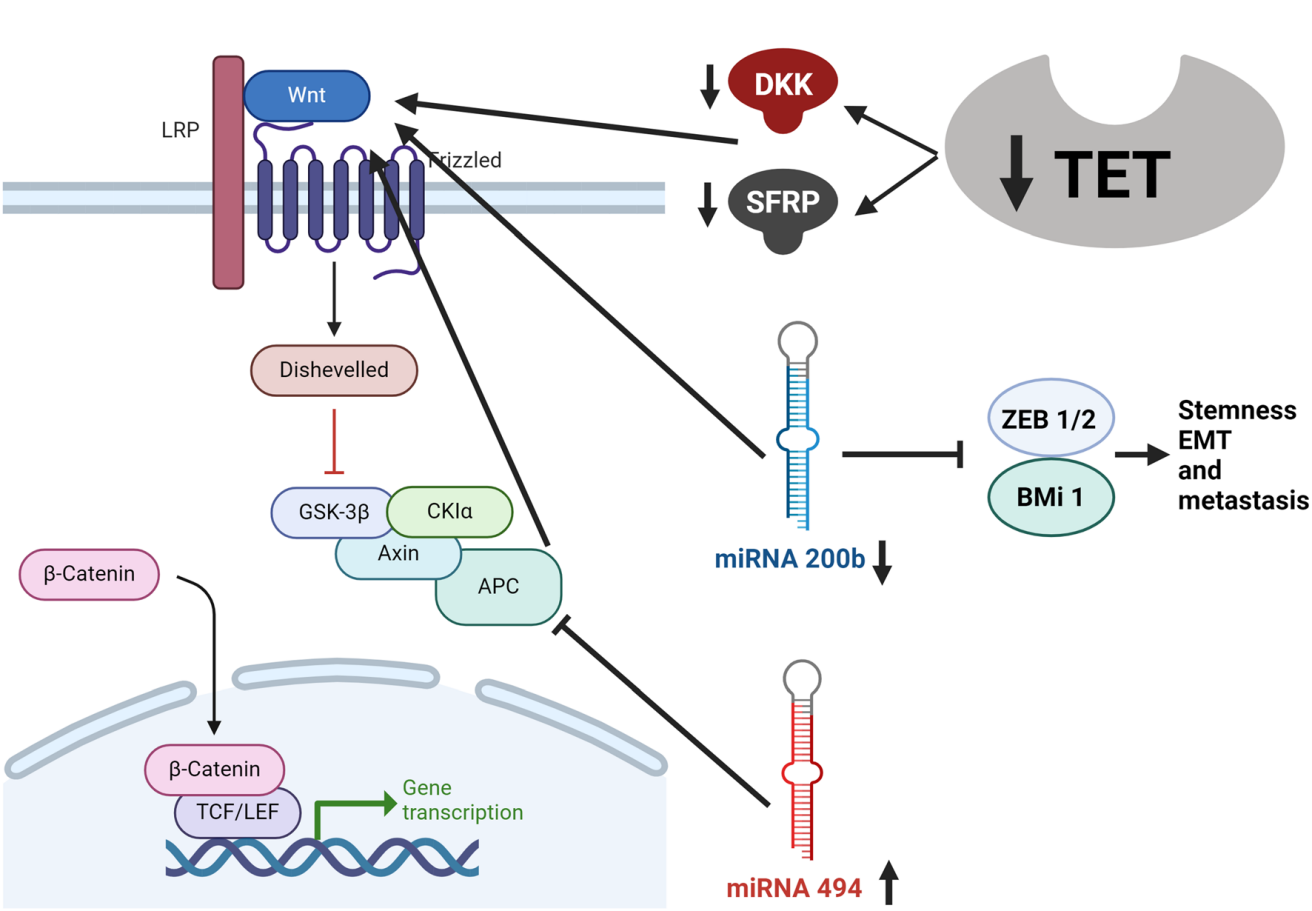
The Proposed crosstalk model between TET1, WNT, mir200b, and miR494 in CRC progression. The reduction in TET family enzymes leads to the demethylation of DKK and SFPR genes, causing disruption of the WNT pathway in cancer. In addition, the decline in TET enzymes is also responsible for the epigenetic inactivation of miR-200b following the reduction in 5hmC levels. This dysfunction of miR-200b triggers the WNT signaling pathway, epithelial-mesenchymal transition (EMT), and metastasis

MiR-200 b is a critical player in suppressing CRC and is often silenced by DNA hypermethylation. Previous studies have shown that the miR-200 family is a target of mutilation, leading to its silencing due to DNA hypermethylation in various cancers [18]. We assessed the expression levels of miR-200 in polyp and tumor samples and their respective adjacent normal tissues. The results showed a significant decrease in the expression of the CRC tissue samples compared to adjacent normal tissue (*P* = 0.008) (Fig. 3). This methylation-dependent silencing aligns with our discovery of reduced miR-200b expression in CRC tissues. The down-regulation of TET1 is likely responsible for silencing miR-200b. MiR-200b regulates critical signaling pathways involved in CRC development, particularly the Wnt signaling pathway [18] (Fig. 4). However, our data indicates that while miR-200b is downregulated in CRC tissue, no significant downregulation was observed in polyp samples. We suggest further evaluation in future studies.

## Conclusions

Our study is the first to report reduced TET1 expression in polyps, thus revealing the new possibilities of its involvement in the early stage of CRC development. Notably, 5-aza increased TET1 expression and decreased Wnt1 expression in CRC cell lines. The upregulation of TET1 may be the result of the inhibition of DNA methyltransferase caused by 5-aza, which brings more substrate for TET1 and enhances its activity.

Furthermore, the expression of miR-200b and miR-494, which play a role in the Wnt pathway and impact on TET1 and Wnt1, was assessed in both polyp and CRC tissue. However, further studies are needed to elucidate the precise mechanisms underlying TET1 and Wnt signaling in CRC and to explore their potential as diagnostic and therapeutic targets. These findings may have important implications for developing novel therapeutic strategies for CRC.

## Data Availability

No datasets were generated or analysed during the current study.

## Abbreviations

Wnt1: Wingless-related integration site
CRC: Colorectal cancer
TET: Ten-eleven translocation
5-aza: 5-azacitidine
APC: Adenomatous polyposis coli
